# Autosomal Resequence Data Reveal Late Stone Age Signals of Population Expansion in Sub-Saharan African Foraging and Farming Populations

**DOI:** 10.1371/journal.pone.0006366

**Published:** 2009-07-29

**Authors:** Murray P. Cox, David A. Morales, August E. Woerner, Jesse Sozanski, Jeffrey D. Wall, Michael F. Hammer

**Affiliations:** 1 ARL Division of Biotechnology, University of Arizona, Tucson, Arizona, United States of America; 2 Department of Mathematics, University of Arizona, Tucson, Arizona, United States of America; 3 Institute for Human Genetics and Department of Epidemiology and Biostatistics, University of California San Francisco, San Francisco, California, United States of America; University of Wisconsin, United States of America

## Abstract

**Background:**

A major unanswered question in the evolution of *Homo sapiens* is when anatomically modern human populations began to expand: was demographic growth associated with the invention of particular technologies or behavioral innovations by hunter-gatherers in the Late Pleistocene, or with the acquisition of farming in the Neolithic?

**Methodology/Principal Findings:**

We investigate the timing of human population expansion by performing a multilocus analysis of≥20 unlinked autosomal noncoding regions, each consisting of ∼6 kilobases, resequenced in ∼184 individuals from 7 human populations. We test the hypothesis that the autosomal polymorphism data fit a simple two-phase growth model, and when the hypothesis is not rejected, we fit parameters of this model to our data using approximate Bayesian computation.

**Conclusions/Significance:**

The data from the three surveyed non-African populations (French Basque, Chinese Han, and Melanesians) are inconsistent with the simple growth model, presumably because they reflect more complex demographic histories. In contrast, data from all four sub-Saharan African populations fit the two-phase growth model, and a range of onset times and growth rates is inferred for each population. Interestingly, both hunter-gatherers (San and Biaka) and food-producers (Mandenka and Yorubans) best fit models with population growth beginning in the Late Pleistocene. Moreover, our hunter-gatherer populations show a tendency towards slightly older and stronger growth (∼41 thousand years ago, ∼13-fold) than our food-producing populations (∼31 thousand years ago, ∼7-fold). These dates are concurrent with the appearance of the Late Stone Age in Africa, supporting the hypothesis that population growth played a significant role in the evolution of Late Pleistocene human cultures.

## Introduction

Reconstructing the timing and magnitude of changes in human population size is important for understanding the impact of climatic fluctuation, technological innovation, natural selection, and random processes in the evolution of our species. With census population sizes estimated to be only in the millions during most of the Pleistocene [1], [2], it is obvious that human population size has increased dramatically towards the present. A major unanswered question is whether expansion began with hunter-gatherer groups, perhaps as a result of the invention of particular technologies or behavioral innovations, or much more recently with the advent of agriculture [3]. Early mtDNA studies suggested that humans experienced a burst of population growth between 30 and 130 thousand years ago (kya)—well before the start of agriculture [4]. More recent results have extended the timeframe for sub-Saharan African growth to 213–12 kya, depending in part on mtDNA haplogroup [5], [6]. However, it is populations—not haplogroups—that are subject to growth, and many present-day hunter-gatherer groups, including those in Africa, do not exhibit any mtDNA signal of demographic expansion at all [7]. On the other hand, Y chromosome sequence data are compatible with a model of constant size for both hunter-gatherer and farming populations in Africa [8]. Autosomal microsatellites tend to indicate an early (pre-Neolithic) start to population growth, but there is disagreement among studies on the time of expansion and whether or not the expansions involved African populations [9], [10]. Zhivotovsky et al. [11] examined a large autosomal microsatellite dataset in 52 worldwide populations and concluded that African farmers, but not hunter-gatherers, exhibit the signal of population growth. Unfortunately, inferences of demographic parameters based on the above mentioned loci may be unreliable due to the possible confounding effects of natural selection or evolutionary stochasticity (for the haploid loci), or uncertainty in our understanding of mutation rates or the underlying mutation process (for mtDNA and microsatellites) [1], [3].

A more reliable source of information regarding past population size change comes from multilocus nuclear sequence studies [12]. Once polymorphism data from multiple X-linked and autosomal loci began to appear, clear discrepancies with inferences based on both mtDNA and microsatellites emerged [13], [14], [15]. For example, most non-African populations tend to have positive Tajima's *D* values—reflecting possible contractions in N_e_—while most African populations tend to have only slightly negative values [16], [17]. Indeed, the largest re-sequencing study to date that targets unlinked autosomal noncoding regions finds that patterns of neutral polymorphism in non-African populations reject the standard constant size model, and are most compatible with a range of bottleneck models invoking a large reduction in effective population size (N_e_) some time after the appearance of modern humans in Africa [18]. In contrast, data from the sole African population examined, the Hausa of Cameroon, were compatible with demographic equilibrium, as well as with a set of recent population expansion models.

In this paper, we expand upon the work of Voight et al. [18] by analyzing a re-sequencing dataset comprised of 20 independently-evolving autosomal noncoding regions in 7 human populations [19]. Our sub-Saharan African populations include the San from Namibia, Biaka from the Central African Republic, Mandenka from Senegal, and Yorubans from Nigeria. Our multilocus analysis, which focuses on two summary statistics with power to detect population growth (Tajima's *D* and Rozas' *R_2_*), follows a two-step approach. We employ a simulation-based method to test the hypothesis that populations experienced exponential growth after a period of constant size. When the hypothesis cannot be rejected, we then fit parameters of this two-phase growth model to our data using approximate Bayesian computation. As in previous studies, we find that the non-African data are not consistent with a simple growth model. On the other hand all four sub-Saharan African samples fit the two-phase growth model, and we are able to infer a range of onset times and growth rates for each population. We sample sub-Saharan African populations that practice different subsistence strategies and then ask whether the inferred signals of population growth are shared between, or specific to, food-gathering or food-producing groups.

## Results

### Patterns of sequence variation

Some basic summaries of the data, including measures of nucleotide diversity (*θ_w_*, *θ_π_*, *η_1_*) and the frequency spectrum of segregating mutations (Tajima's *D*, Rozas' *R_2_*), are provided in **Table 1**. As reported previously [19], we find that mean autosomal values of Tajima's *D* are slightly negative in our sub-Saharan African populations (−0.243, −0.350 and −0.139 for the San, Biaka and Mandenka, respectively). The Yoruban results, which are based on a larger sample (*n* = 94 individuals) with more loci (*n* = 31; albeit with fewer sequenced sites per locus), show a similar mean value of Tajima's *D* (−0.287). The proportion of sites with singleton mutations (i.e., *η_1_*/*S*) ranged from 19% in the Yorubans to 29% in the Biaka (mean = 26%). In comparison, non-African populations exhibit a positive mean value of Tajima's *D* (0.302) [19], a higher mean value of Rozas' *R_2_* (0.142), and a lower mean proportion of singletons (19%) (data not shown). Depressed values of Tajima's *D* and Rozas' *R_2_*, coupled with an elevated proportion of singletons, is suggestive of population growth.

**Table 1:** Mean summary statistics for 4 African populations.

Population	N	Loci	l	S	η_1_	θ_W_ (%)	θ_π_ (%)	Tajima's D	Rozas' R_2_
SAN	19.5	20	113	501	160	0.134	0.126	−0.243	0.124
BIA	28.0	20	113	574	172	0.134	0.121	−0.350	0.110
MAN	28.2	20	113	539	147	0.125	0.120	−0.139	0.117
YOR	187.4	31	61	466	85	0.132	0.116	−0.287	0.076

*N*, number of chromosomes.

*l*, length of sequence (kb).

*S*, number of segregating sites.

### Do the Data Fit a Two-Phase Growth Model?

**Figure 1:**
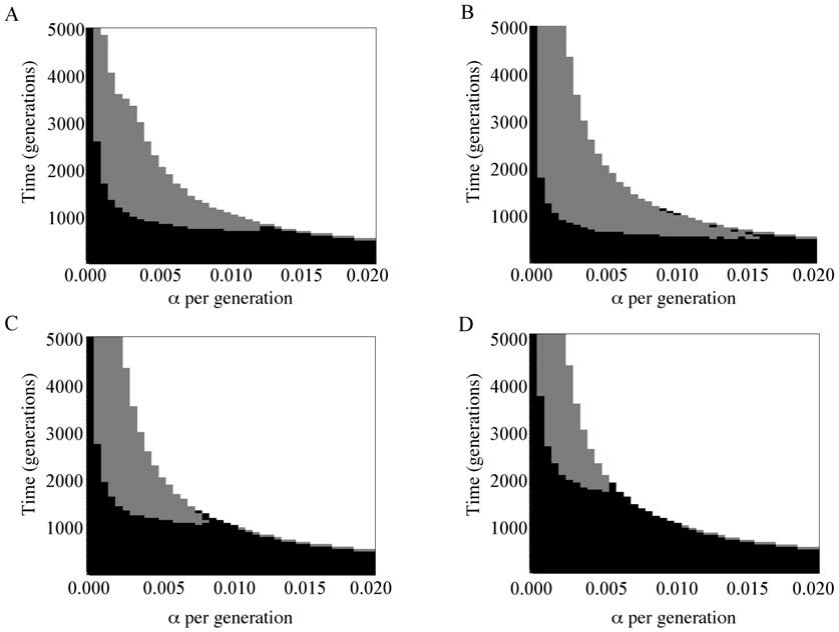
Times of onset of growth in generations (*y*-axis) and growth rates per generation (*x*-axis) inferred from autosomal data under the two-phase growth model using the mean and variance of Rozas' *R_2_* across loci for (A) Biaka, (B) San, (C) Mandenka and (D) Yorubans. White indicates both mean and variance rejected at the 5% level; grey indicates either mean or variance rejected; black indicates neither mean nor variance rejected.

We tried to reject a series of two-phase growth models for each of the six populations reported in Wall et al. (2008) separately using Tajima's *D*, Rozas' *R_2_*, and the variances of these two summary statistics. Tajima's *D* and Rozas' *R_2_* consistently give similar probability values with the hypothesis-testing method developed by Pluzhnikov et al. [20], and thus, subsequent results are presented only for Rozas' *R_2_*. In contrast to the three non-African populations (data not shown), we find that the two-phase growth model cannot be rejected for a range of *τ* and *α* when applied to the African autosomal data (**Figure 1A–C**). A range of growth models could not be rejected (i.e., *P*>0.05) for all of our African populations, and we observed that multi-locus *P*-values attained their maxima strictly away from a growth rate of zero. This suggests that the data better fit a two-phase growth model than constant population size. Similar results were obtained for the larger Yoruban sample (**Figure 1D**).

### Inferring Parameters of the Two-Phase Growth Model

**Figure 2:**
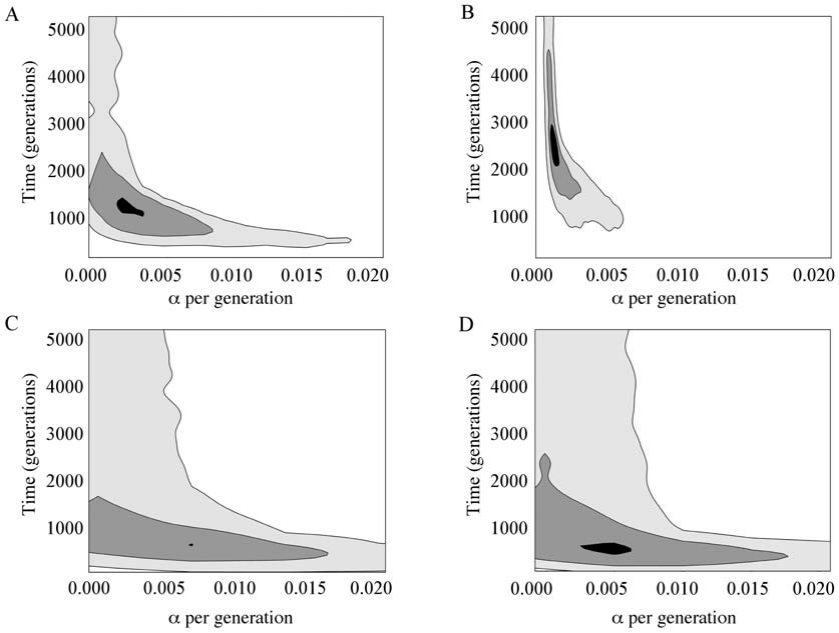
Times of onset of growth in generations (*y*-axis) and growth rates per generation (*x*-axis) inferred from autosomal data under the two-phase growth model using ABC on Rozas' *R_2_* and *S* of all loci individually for (A) Biaka, (B) San, (C) Mandenka and (D) Yorubans. The maximum likelihood estimate falls within the black-filled region, with black, dark gray, and light gray shading indicating 10%, 50%, and 95% contour lines, respectively. Unshaded regions were rejected at the 5% level.

To infer the range of growth parameters consistent with the data, we applied approximate Bayesian computation (ABC) to the autosomal sequences obtained from our three African populations (**Figure 2**) (See **Supplementary Text S1 and Figures S1 and S2** for validation of the ABC method employed here). We infer median growth rates, *α*, of 8.5×10^−4^/generation (95% credible region: 5.9×10^−5^–7.4×10^−3^), 1.1×10^−3^ (1.8×10^−5^–2.1×10^−2^), and 5.2×10^−4^ (5.9×10^−6^–6.2×10^−2^), for the San, Biaka and Mandenka, respectively (**Table 2**). On average, these rates reflect 14-, 11- and 9-fold growth from ancestral population sizes (**Table 2**). Median times since the onset of population growth are 1,863 (513–6,625), 1,027 (97–6,656), and 901 (38–6,497) generations ago, for the San, Biaka and Mandenka, respectively. Given a generation interval of 28 years [21], these values correspond to chronological dates of 52, 29 and 25 thousand years ago (or 37, 21 and 18 kya if we assume a 20-year generation interval). We obtain similar results with our larger Yoruban dataset. We infer a growth rate of 1.7×10^−4^ per generation (4.3×10^−6^–6.6×10^−2^), and a time of onset of growth at 1,280 (28–6,780) generations ago (or 36 kya), and 5-fold growth from ancestral size (**Table 2**). Our 3-dimensional 95% credible region, as approximated by a scaled 10×10×10 grid over the posterior of *N_A_*, *N_0_* and *τ*, returns Bayes' factors (*K*) ranging from 57 to 70 (**Table S2**). Measured against the Jeffreys' [22] scale, this indicates very strong support for our posterior distributions and the demographic models we infer from them.

**Table 2:** Growth parameters (means and 1-dimensional 95% confidence intervals) for 4 African population based on Rozas' *R_2_*.

	τ(gen)	τ(kya)	α(×10^−3^)	N_A_ (×10^3^)	N_0_ (×10^3^)
SAN	1,860 (513–6,630)	52 (14–186)	0.85 (0.059–7.4)	11.2 (10.2–12.2)	148 (16–811)
BIA	1,030 (97–6,660)	29 (3–186)	1.1 (0.018–21)	10.7 (9.7–11.6)	119 (12–770)
MAN	900 (38–6,500)	25 (1–182)	0.52 (0.0059–62)	10.8 (9.7–12.2)	94 (11–679)
YOR	1,280 (29–6,780)	36 (1–190)	0.17 (0.0043–66)	11.9 (10.2–14.2)	63 (11–552)

*τ*, time of onset of growth (in generations and thousands of years, respectively).

*α*, rate of growth (per generation).

*N_A_*, ancestral effective population size.

*N_0_*, modern effective population size.

### Size Changes Inferred under the Isolation-with-Migration Model

Modern and ancestral effective population sizes were also inferred for the same 20-locus autosomal dataset under the isolation-with-migration model implemented by Jody Hey and colleagues [23]. Marginal posterior densities for population split times and split proportions could not be inferred with accuracy [24]. However, assuming an equal division of ancestral populations, the San, Biaka and Mandenka are inferred to have grown 5-, 4- and 7-fold from ancestral population sizes. These growth rates are lower than estimates obtained using ABC, and are suggestive of faster growth rates in the food-producing Mandenka compared with our hunter-gather groups, the San and Biaka, *contra* our findings based on ABC. Note, however, that our ABC and IM results are not strictly comparable because they employ different demographic models; in particular, the isolation-with-migration model incorporates the effects of past gene flow and shared ancestry among populations. More importantly, both analyses suggest that all three sub-Saharan African populations have not maintained constant population size, but have instead experienced some amount of growth.

## Discussion

Our understanding of population size changes in human prehistory has improved as our genetic datasets and analysis methods have become more sophisticated. Early studies of the pairwise mismatch distribution in mitochondrial DNA (mtDNA) suggested dramatic increases in population size between 110 and 70 kya in sub-Saharan Africa [25], [26]. More recent coalescent studies have also favored 50- to 100-fold growth occurring between 213 and 12 kya [5], [6]. Conversely, modern surveys of nuclear sequence variation at unlinked loci have not provided clear evidence for rapid population growth from small ancestral size. For example, African populations usually exhibit slightly negative Tajima's *D* values, while non-African populations tend to have positive Tajima's *D* values [13], [14], [16], [17], [18], [27]. Different patterns of polymorphism in African and non-African populations have been interpreted as reflecting a history of bottleneck(s) in the ancestry of non-Africans [20], [28], [29], [30], [31]. Therefore, the question of when anatomically modern human populations began to expand in size is better addressed in sub-Saharan African populations because more recent demographic events likely obscure signals of population growth in the ancestors of non-African groups [28]. Bottlenecks, in particular, can mask the effects of earlier, as well as later, population growth.

However, thus far, very few surveys of nuclear DNA sequence variation have been performed in sub-Saharan African populations, and interpretations drawn by existing studies have been complicated by the different populations and loci analyzed, the kinds of analyses performed, and the different growth models assumed. The earliest studies considered only the few existing nuclear sequence data available in the literature at the time, and explored only a small set of growth model parameters [3]. Later studies adopted a more explicit hypothesis-testing framework, but focused on only a single African population. For instance, Pluzhnikov et al. [20] analyzed a large resequence dataset of noncoding autosomal regions for the Hausa of Cameroon (a food-producing group). They determined that while observed summaries of the site frequency spectrum did not statistically reject a null model of constant size, they were consistent with a range of alternative growth models. Consequently, Voight et al. [18] turned to a goodness-of-fit approach to determine better estimates of the time of onset of growth and the growth rate in the Hausa. By generating approximate likelihoods for the mean of observed summary statistics over a grid of parameter values, they determined that the Hausa best fit a growth model beginning ∼1,000 generations ago with a per-generation growth rate *α* of 0.75×10^−3^. Assuming a generation time of 25 years, this corresponds to an overall ∼2-fold growth rate from ancestral to modern size beginning ∼25 kya.

Here, we extend these sorts of analyses to a greater range of African populations: two hunter-gathers, the San of Namibia and the Biaka of the Central African Republic; and two food-producers, the Mandenka of Senegal and the Yorubans of Nigeria. All four groups show depressed values of Tajima's *D* and Rozas' *R_2_* coupled with a high proportion of singleton mutations (**Table 1**). These patterns of sequence polymorphism are suggestive of population growth. We therefore tested our multilocus African dataset to determine whether we could reject models of population growth, and adopted the best aspects of previous hypothesis-testing and inference approaches. We first employed hypothesis-testing to determine, by coalescent simulation, whether a range of growth models could be rejected in favor of constant size using the method pioneered by Pluzhnikov et al. [20]. When growth could not be rejected, we fitted parameters of the two-phase growth model to our data using approximate Bayesian computation (**Table 2**). Thus, we conditioned simulations on each locus individually (including mutation and recombination rates), and explored a continuous range of parameter values rather than restricting our search to a set of predetermined grid coordinates. We note that the overall trend of both our hypothesis-testing and ABC results are strongly concordant (**Figures 1** **and** **2**).

All of our African populations best fit models with relatively low population growth (∼10-fold) beginning in the late Pleistocene (∼36 kya). Even with ∼112-kb of sequence data per individual, a large range of growth models are consistent with our 95% credible regions for *τ* and *α*. We cannot, for instance, statistically distinguish different rates and times of growth among our four sub-Saharan African samples. However, our hunter-gather populations show a tendency towards slightly older and stronger growth (∼41 kya, ∼13-fold) than our food-producing populations (∼31 kya, ∼7-fold). Furthermore, we detect a strongly negative, non-linear association between *τ* and *α* (Spearman's correlation, *ρ* = −0.91 to −0.93, all *P*≪0.001). This effect, which has been identified previously [20], implies that sequence data from our four African populations are consistent either with weaker growth beginning earlier in the Late Pleistocene, or with stronger growth commencing more recently. Interestingly, we can reject an onset of population growth for the San during the Holocene (lower 95% confidence bound = 14 kya), and therefore, growth in this population is not linked to the development of agriculture. Although we cannot reject an onset of growth associated with agriculture for the Biaka, Mandenka and Yorubans, our best fitting models do not favor this interpretation. Indeed, the limited size of our dataset gives us more power to infer older rather than more recent growth [28].

We see little effect from the increased size of the dataset obtained for Yorubans. Even though we increased both the number of samples (from 16 to 90 individuals) and the number of loci (from 20 to 31), estimates of the rate and timing of growth are comparable to those inferred for the Mandenka, and our 95% credible region is not appreciably smaller. This is interesting given that, under a model of population growth, expected values of Tajima's D depend to some extent on sample size [32], [33]. With regard to the small increase in the number of loci in our Yoruban dataset, recent power analyses by Adams and Hudson [28] suggest that orders of magnitude more data may be necessary to obtain growth model parameters with substantially greater accuracy, especially in models involving recent growth. Furthermore, the modern effective sizes we infer – on the order of 10^5^ – are much smaller than regional census sizes. This discrepancy partly reflects the fact that effective size is not a simple proxy for census size. However, another explanation also seems likely: under a model of exponential growth, the bulk of the population increase is weighted towards the present, and for the aforementioned reasons [28], we are not likely to capture the effects of substantial increases population size in modern times.

Although population growth seems like a reasonable demographic model for human groups on non-genetic grounds [1], [2], [34], humans have likely experienced both population growth and population structure at some time in the past. The question is whether and to what extent either or both of these aspects of population history left a signature on patterns of variation. To explore the effects of alternate models of population structure on patterns of genetic variation, we use a coalescent simulation approach. In particular, we examine how Tajima's *D* and Rozas' *R_2_* respond under models incorporating low-frequency gene flow in a structured population, recent admixture, and cryptic population structure (see **Supplementary Text S1, Figures S3–S5**). We assume a two-deme splitting model with i) a constant low level of gene flow (i.e., 0≤*Nm*≤1) [24], ii) a single admixture event occurring ∼3 kya (i.e., corresponding to the Bantu expansion), and iii) population structure collapsing ∼150 years ago (i.e., cryptic population structure). All of these processes produce very slight reductions in Tajima's *D* and Rozas' *R_2_*, but the mean deviations never exceed 0.27 and 0.011, respectively. To put these values in perspective, such deviations represent no more than 10% and 12% of the variance naturally observed for Tajima's *D* and Rozas' *R_2_* under the corresponding standard neutral models with no gene flow, admixture, or cryptic population structure. Although these confounding factors may have caused our growth estimates to appear slightly older or stronger than they actually are, their effects are minor. Similarly, biases in our estimates of per-locus mutation and recombination rates are unlikely to have major effects on our inferences. For instance, elevated recombination would lead to a lower variance of Tajima's *D* and Rozas' *R_2_*, which would return growth estimates with less uncertainty, while elevated mutation rates would shorten our time frames, and hence return younger growth estimates.

Estimates of growth rates under the isolation-with-migration model, which simultaneously accounts for population structure and gene flow, are consistent with our inference of an increase in the effective size of sub-Saharan African populations. Although growth rates are lower than suggested by ABC, we still infer that African populations experienced ∼5-fold growth from ancestral sizes. While a simple two-phase growth model is too simplistic to fully describe African population history, it is interesting to note that a more complex model incorporating an ancient bottleneck (i.e., prior to the onset of population growth) does not fit African resequencing data [18], [28]. This is in marked contrast to the large reduction in population size that the same studies inferred for non-Africans. We therefore suggest that our growth estimates genuinely reflect a substantial increase in effective size among sub-Saharan African populations beginning in the Late Pleistocene. However, we note that these inferences could be complicated by other forms of population structure not accounted for in our models.

While some authors have speculated that human populations underwent sudden expansions in population size in response to dramatic climatic events, technological inventions, or behavioral changes that took place earlier than 50 kya [35], [36], [37], [38], our data are more consistent with a model of exponential growth beginning after 50 kya, but certainly before the Holocene. This is concordant with several archaeological indicators showing long-term increases in population density in the Upper Paleolithic and Late Stone Age, including increased small-game exploitation, greater pressure on easily collected prey species like tortoises and shellfish, and more intense hunting of dangerous prey species [39], [40], [41], [42]. We further note that much of the literature pointing to sudden increases in effective population size beginning earlier in the Pleistocene in sub-Saharan Africa (i.e., 110–70 kya) is based on mtDNA data, which tends to show unimodal mismatch distributions and a skew in the frequency distribution towards rare alleles in many African farming and non-African populations [25], [26]. However, this mtDNA signal of demographic expansion is typically absent from samples of African hunter-gatherers [25]. Our autosomal data provide a very different picture of more recent (and moderate) population growth in both sub-Saharan African hunter-gatherers and food producers. Preliminary simulations (**Supplementary Text S1**) indicate that a model of population growth similar to that tested here does not result in elevated values of Tajima's *D* and Rozas' *R_2_* for mtDNA as a result of its smaller effective population size relative to the autosomes [13]. On the contrary, the four-fold smaller *N_e_* of mtDNA means that it should reflect population growth more prominently (**Figures S6** and **S7**). Consequently, mtDNA data may not accurately tell us when and to what extent human populations expanded, either as a result of evolutionary stochasticity (which introduces uncertainty when making inferences based on a single haploid locus), or as a result of natural selection at functional sites (which would bias patterns of linked neutral variation across the mtDNA genome). We specifically avoid these issues by considering multiple, independent, neutral regions from the autosomes.

In sum, the ∼1000-fold increase in human population size that has taken place over the last 10 kya (e.g., from ∼6 million to over 6 billion people today) [2] is unlikely to be detectable with current resequencing data [28]. The finding that autosomal resequencing data from all sub-Saharan African populations so far tested (n = 5) contain a signal of exponential size increase beginning in the Late Stone Age [18], [20], [28] is concordant with archaeological data showing intensification in the number of LSA sites on the African landscape, an increased abundance of blade-based lithic technologies, and enhanced long-distance exchange after 50 Kya [39], [40], [41], [42]. Interestingly, there is mounting evidence that many of the individual elements of complex behavior first appear earlier in the Middle Stone age, 70–100 Kya [41]. This suggests that the demographic effects manifest in these indicators of modern culture were felt only sporadically in the MSA, and that they did not become the general condition until the LSA, coincident with the significant population growth that is detectable in the autosomes of contemporary sub-Saharan Africans.

## Methods

### Population Samples and Sequenced Loci

We have previously reported genomic data for three non-African (French Basque, Chinese Han and Melanesians) and three sub-Saharan African (San, Biaka and Mandenka) populations [19]. Approximately 16 individuals were sampled for each population, with the exception of the San (**Table 1**). For each individual, we re-sequenced a total of ∼6 kb from each of 20 autosomal intergenic regions (i.e., a total of ∼112 kb for each individual). We employed a locus trio design whereby we sequenced 3 fragments of ∼2 kb spaced evenly across an ∼20 kb region (see Wall et al. 2008 for details). To increase our power to detect population growth, we also sequenced a similar set of autosomal intergenic regions in a much larger sample of Yorubans (*n* = 94). In this case, only a single fragment (∼2 kb) of the locus trio was sequenced in each of 31 autosomal regions. These loci included some, but not all, of the 20 autosomal regions sequenced for the Wall et al. (2008) dataset (**Table S1**). Yoruban DNA samples were obtained from the NHGRI collection at the Coriell Institute for Medical Research (i.e., the Yorubans from Ibadan, Nigeria panel used in the HapMap project).

### Summary Statistics

We focused on three summary statistics: the number of segregating sites *S*, which controls for the population mutation rate *θ* ( = 4*N_e_μ*); Tajima's *D* [43], which summarizes the normalized difference *θ_π_* - *θ_w_*; and Rozas' *R_2_* [44], which captures the normalized difference between *θ_π_*/2 and the observed number of singletons (*η_1_*). The expectation of Tajima's *D* is close to zero under a Wright-Fisher model with no population growth, whereas the expectation of Rozas' *R_2_* is zero under a Wright-Fisher model with very strong population growth (i.e., a perfectly star-like genealogy). All of our summary statistics were calculated with *libsequence* [45], to which we added a C++ function that calculates Rozas' *R_2_* (code available on request). The term *U_i_* in equation 1 of Ramos-Onsins and Rozas [44] does not unambiguously define singletons as derived mutations from the unfolded site frequency spectrum. Because full outgroup sequences were generated for this study, we applied this unfolded definition here.

### Demographic Models

We initially considered a single-deme two-phase growth model in which an ancestral population of size *N_A_* grew exponentially at time *τ* to its modern effective size *N_0_*. This model, which assumes an ancestral phase of constant population size followed by a more recent phase of exponential population growth, has three parameters: the ancestral population size *N_A_*, the time of the onset of growth *τ*, and the population growth rate *α* (where *α*≥0). The population size *N_t_* at generation *t* since the present is given by
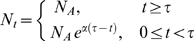
(1)


This parameter space can be reduced by one dimension if the ancestral population size is calculated such that, given *τ* and *α*, the expected number of segregating sites matches the observed data [20]. We applied this approximation in our hypothesis-testing phase. Later, we considered other demographic models that might be confounded with population growth, such as gene flow, admixture and substructure.

### Hypothesis Testing

First, we explored the data to see whether we could reject two-phase population growth in favor of constant population size [20]. We simulated sequence data for each population across a grid of values for *τ* and *α*, including constant size (i.e., *τ* = *α* = 0), using the *n*-coalescent model implemented in *ms* [46]. For each simulated locus, we filtered the data to mimic our trio-based sequencing design, and calculated *S*, Tajima's *D* and Rozas' *R_2_*. By repeating this process 10^4^ times, we obtained summary statistic distributions from which we could calculate means and variances across loci. A multi-locus *P*-value was determined by comparing these distributions to observed values, where *P* is the fraction of simulated summary values that lie further from the mean than our observed value (i.e., similar to a two-tailed test). A *P*-value of one implies that observed values are exactly as expected under the simulated model. *P*-values were calculated separately for the mean and variance, and for each summary statistic. We rejected all models for which *P*<0.05.

### Demographic Parameter Inference

For populations that failed to reject variants of a two-phase growth model, we inferred growth parameters with greater resolution using approximate Bayesian computation (ABC) [47]. Because the derivation and calculation of coalescent likelihoods can be prohibitively difficult, ABC replaces the full dataset with one or more summary statistics. The demographic state-space 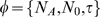 was explored by comparing the statistic *λ* observed for a given locus with its expectation *Λ* under a randomly chosen demography *φ′*. Demographic parameter sets *φ′* that produce simulated data with a mean summary statistic close to the observed value reflect best estimates of the true demography.

Demographic parameters for effective sizes were drawn from random log-uniform priors: *N_A_* ranged from 10^3^ to 5×10^4^, and *N_0_* ranged from 10^3^ to 10^5^. The time of onset of growth, in years, was drawn from a random uniform prior: *T_years_* ∈ *Unif*(1, 2×10^5^). *N_0_* was constrained, such that *N_0_*≥*N_A_*, to ensure that all genealogies eventually coalesced. Mutation and recombination rates, sequence lengths, and sample sizes were conditioned on each locus individually. Mutation rates were estimated using the mean divergence between all human sequences and *Pan* outgroups divided by a *Homo*/*Pan* divergence time of 6×10^6^ years. Recombination rates were inferred directly from sequence data using the algorithm implemented in *LDhat* [48]. To convert generation estimates into chronological dates, we assumed a generation interval for modern humans of 28 years [21].

We employed the following ABC algorithm: (1) choose a summary statistic *Λ* and calculate its value *λ* for the empirical data set; (2) choose a tolerance *δ*; (3) pick a random set of demographic parameters *φ′* from the prior distribution of *φ*; (4) simulate 10^4^ coalescent datasets for a given locus under the chosen model; (5) compute the distribution of the summary statistic *Λ* from the simulated data; (6) repeat steps 4–5 for all loci (*n* = 20) and all summary statistics (*n* = 2); (7) standardize each *Λ* and *λ* to *Λ′* and *λ′*, respectively (see below), and calculate the standardized distance *d*(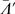, *λ′*) for all loci and all summary statistics; (8) repeat steps 3–7 until *k* ( = 10^5^) replicates are obtained; and finally (9) reject all *φ′* for which *d*>*δ*. The distance *d* was defined as the *n*-space Euclidean metric
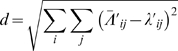
(2)calculated across all loci *i* and all summary statistics *j*. The tolerance *δ* was taken as the first percentile of the ranked distribution of distances 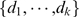.

This approach differs slightly from previous ABC algorithms by standardizing the distributions of summary statistics. The distribution of *Λ* is normalized such that *Λ′* = (*Λ*-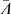)/*σ*(*Λ*), which subsequently has mean of zero and standard deviation of one regardless of the original distribution of *Λ*. This ensures equal weighting of summary statistics with quite different numerical distributions, such as *S* (zero and positive integers), Tajima's *D* (all real numbers), and Rozas' *R_2_* (zero and positive real numbers).

### The Isolation-with-Migration Model

Finally, modern and ancestral effective population sizes were also inferred under the isolation-with-migration model implemented in the software IM v. 31 July 2006 [23], [49], [50]. Unlike the methods described above, the isolation-with-migration model compares populations in pairwise fashion; shared ancestry and gene flow are therefore accounted for. Note that growth in the isolation-with-migration model is defined as starting when the two populations split, rather than allowing a phase of constant population size followed only later by population growth. Thus, our IM results may be informative about growth rates, but not the time of onset of growth.

## Supporting Information

Text S1 (0.03 MB DOC)

Table S1 Genomic loci analyzed in this study.
(0.04 MB DOC)

Table S2 Bayes factors for 3-dimensional 95% credible region inferred by ABC.
(0.02 MB DOC)

Figure S1 Validation results for the ABC procedure. Comparison of median values from joint posterior distributions for times of onset of growth (left column) and growth rates (right column). Median values (dotted vertical lines) inferred from 5 simulated datasets, each comprising 20 autosomal loci, are compared with known model parameters (solid vertical lines). ABC was employed with Rozas' R2 (upper row), Tajima's D (middle row), and both summary statistics jointly (bottom row). The number of segregating sites S was used to constrain θ in all cases. See text for details.
(0.14 MB DOC)

Figure S2 Validation results for the ABC procedure. Comparison of 95% credible region of joint posterior distributions for times of onset of growth (left column) and growth rates (right column). Lower confidence bounds, median values and upper confidence bounds inferred from 5 simulated datasets, each comprising 20 autosomal loci, are compared with known model parameters (solid vertical lines). ABC was employed with Rozas' R2 (upper row), Tajima's D (middle row), and both summary statistics jointly (bottom row). The number of segregating sites S was used to constrain θ in all cases. See text for details.
(0.18 MB DOC)

Figure S3 Effect of gene flow on Rozas' R2 and Tajima's D in a two-deme splitting model with asymmetric migration. Circles indicate mean values; dotted lines indicate 95% credible regions.
(0.09 MB DOC)

Figure S4 Effect of Bantu admixture (∼3 kya) on Rozas' R2 and Tajima's D in a two-deme splitting model. Circles indicate mean values; dotted lines indicate 95% credible regions.
(0.10 MB DOC)

Figure S5 Effect of recent cryptic structure (∼150 years ago) on Rozas' R2 and Tajima's D in a two-deme splitting model. Circles indicate mean values; dotted lines indicate 95% credible regions.
(0.10 MB DOC)

Figure S6 Time progression showing the expectation of Rozas' R2 following onset of growth. Haploid loci (circles) respond more quickly to growth (i.e., values of Rozas' R2 approaching zero) than autosomal loci (triangles).
(0.08 MB DOC)

Figure S7 Time progression showing the expectation of Tajima's D following onset of growth. Haploid loci (circles) respond more quickly to growth (i.e., negative values of Tajima's D) than autosomal loci (triangles).
(0.08 MB DOC)
