# Supplementary material for: Autosomal Resequence Data Reveal Late Stone Age Signals of Population Expansion in Sub-Saharan African Foraging and Farming Populations
Source: PLoS One. 2009 Jul 29;4(7):e6366. doi: 10.1371/journal.pone.0006366 (PMC2712685; doi:10.1371/journal.pone.0006366)
Supplement: Text S1 — (0.03 MB DOC) [file pone.0006366.s001.doc]

**Supplementary Text S1**

**ABC Validation**

The approximate Bayesian computation was validated on a randomly-chosen demography, a single Wright-Fisher deme in which growth began 715 generations ago at a rate, *α*, of 1.3x10^-3^/generation. Five datasets, each comprising 20 autosomal loci, were simulated under this model to mimic the empirical dataset. The ABC procedure was applied to each simulated dataset using the number of segregating sites *S* to constrain the population mutation rate *θ*, and the summary statistics Rozas’ *R_2_* and Tajima’s *D*, both singly and jointly, to infer the timing and rate of population growth. Analyses employing only Rozas’ *R_2_* and *S* were most accurate (**Figure S1**). On average, the median values of posterior distributions for the time of onset of growth underestimated the true value by only 7%, whereas the true value of the growth rate was overestimated by only 7%. Conversely, the use of Tajima’s *D* and *S* underestimated the time of onset of growth by 27%, and overestimated the growth rate by 104%. Rozas’ *R_2_*, Tajima’s *D* and *S* applied jointly underestimated the time of onset of growth by 27%, and overestimated the growth rate by 72%. This result did not change materially when a smaller tolerance threshold *δ* was employed (*i.e.,* 0.1% versus 1%). In fact, the larger tolerance threshold routinely produced more accurate point estimates and smaller confidence intervals for the demographic parameters under study (unpublished data). Because posterior distributions routinely have substantial variance, the 95% confidence intervals reported here are large (**Figure S2**). Nonetheless, the medians of posterior distributions generated using ABC with Rozas’ *R_2_* and *S* are consistently accurate, and this method was applied subsequently to our African population data. It should be recognized that this inference approach is computationally expensive; the results described above required ~25,000 CPU hours to generate and analyze some 300 billion coalescent datasets. Nonetheless, the validation procedure applied here is standard for inference methods of this type (Hey and Nielsen 2004).

**Demographic Processes Confounded with Growth**

To check whether estimates of growth might be conflated with gene flow or admixture, we explored how these factors affect Rozas’ *R_2_* and Tajima’s *D* using coalescent simulation. We sampled from a pure splitting (or phylogenetic) model containing two constant sized demes (N_0_ = 10^4^) that diverged 50 kya (or 1785 generations assuming a 28-year generation interval). We conditioned this model on parameters taken from our empirical dataset: a sample size of 28 autosomal chromosomal copies, and mean values for sequence length, mutation rate and recombination rate. We only sampled individuals from the first deme; the second deme was modeled solely to provide a population source for gene flow and admixture.

Migration

We allowed long-term asymmetric gene flow from deme 2 to deme 1, and varied the population migration rate (*Nm*) from 0 to 1. Effectively, therefore, this system represents an isolation-with-migration model. We found that both Rozas’ *R_2_* and Tajima’s *D* are negatively biased, but the effect is minor (**Figure S3**). The largest deviation is observed when *Nm* = 1. Relative to a model with zero migration (i.e., *Nm* = 0), Rozas' *R_2_* has a maximum mean deviation of 0.0031, while Tajima’s *D* has a maximum mean deviation of 0.075. For comparison, however, Rozas’ *R_2_* has a 95% confidence interval of (0.074, 0.17; i.e., a range of 0.096) and Tajima's *D* has a 95% confidence interval of (-1.3, 1.3; i.e., a range of 2.6) under a model with zero migration. Hence, these maximum deviations represent only ~3% of the variance customary for these summary statistics.

Admixture

Two related models were explored: i) admixture resulting from the expansion of Bantu-speaking agriculturalists, and ii) a reduction in population structure that occurred just prior to recorded history (i.e., recent cryptic population structure). We allowed instantaneous admixture from deme 2 to deme 1 either 107 generations ago (i.e., admixture ~3 kya during the Bantu expansion), or 5 generations ago (i.e., recent, but now forgotten, population mixing ~150 years ago). We allowed the admixture proportion to vary from 0 (i.e., no admixture) to 1 (i.e., complete mixing of the two demes).

We found that both Rozas’ *R_2_* and Tajima’s *D* are negatively biased under a model of the Bantu expansion (**Figure S4**), but the effect is slightly more pronounced with recent admixture (**Figure S5**). Because both models are similar, we only discuss the latter here. The largest deviation was observed with 50% admixture. Relative to a model with zero admixture, Rozas' *R_2_* has a maximum mean deviation of 0.011, while Tajima’s *D* has a maximum mean deviation of 0.27. These maximum deviations represent 12% and 10%, respectively, of the variance that we observe under the model with no admixture.

Population growth causes Rozas’ *R_2_* to approach zero and Tajima’s *D* to become negative. Although gene flow and admixture bias these summaries in the same direction, the effect is relatively weak (note maximum deviations above), and these factors are not sufficient to explain the values of Rozas’ *R_2_* and Tajima’s *D* that we observe in African populations. In the presence of gene flow or admixture (or a combination of both), our inference methods would tend to overestimate the effects of population growth, thus leading us to infer slightly older and stronger growth than actually occurred. This confound is, however, in the wrong direction for a model of growth associated with agriculture, and in any case, the effect is sufficiently weak that our main conclusions remain unaffected.

Finally, we note that outbound migration does not affect estimates of Rozas’ *R_2_* and Tajima’s *D* (i.e., these summaries do not change in deme 2), while symmetric migration produces similar, but less extreme, deviations than we observed for the asymmetric model above (unpublished data).

**Reconciling Different Patterns between Autosomal and Mitochondrial DNA**

To determine whether different compartments of the genome respond similarly to population growth, we modeled a single deme with an ancestral effective size of 10^4^, and allowed it to grow to a modern effective size of 10^5^ (i.e., 10-fold growth) over the course of 2,000 generations (or 50 kya, assuming a 25-year generation interval). We obtained mean values for Rozas’ *R_2_* and Tajima’s *D* for haploid loci (i.e., *θ* = 1*N_A_μ* = 10) and autosomal loci (i.e., *θ* = 4*N_A_μ* = 40) using 10^5^ coalescent simulations at each time point. Under strong population growth, Rozas’ *R_2_* should approach zero and Tajima’s *D* should become increasingly negative. We observe this trend (**Figures S6 and S7**, respectively), but note that haploid loci respond to growth in a more extreme manner than autosomal loci. Our empirical dataset of mtDNA and autosomal genetic sequences do not show this association (data not shown).
