# Supplementary material for: Autosomal Resequence Data Reveal Late Stone Age Signals of Population Expansion in Sub-Saharan African Foraging and Farming Populations
Source: PLoS One. 2009 Jul 29;4(7):e6366. doi: 10.1371/journal.pone.0006366 (PMC2712685; doi:10.1371/journal.pone.0006366)
Supplement: Table S1 — Genomic loci analyzed in this study. (0.04 MB DOC) [file pone.0006366.s002.doc]

**Table S1. Genomic loci analyzed in this study.**

| *Locus* | *Chr* | *Start Base (2004 Coordinates)* | *End Base (2004 Coordinates)* | *2004 UCSC Coordinates* | *2006 UCSC Coordinates* |
| --- | --- | --- | --- | --- | --- |
| 1pMB101 | 1 | 101,746,289 | 101,748,285 | chr1:101746289-101748285 | chr1:101806856-101808852 |
| 1pMB81 | 1 | 81,095,700 | 81,097,555 | chr1:81095700-81097555 | chr1:81156267-81158122 |
| 2pMB6 | 2 | 6,215,789 | 6,217,806 | chr2:6215789-6217806 | chr2:6182642-6184659 |
| 2qMB134 | 2 | 134,434,942 | 134,436,793 | chr2:134434942-134436793 | chr2:134317680-134319531 |
| 3qMB148 | 3 | 148,804,809 | 148,806,741 | chr3:148804809-148806741 | chr3:148804801-148806733 |
| 4pMB31 | 4 | 31,391,076 | 31,393,099 | chr4:31391076-31393099 | chr4:31323905-31325928 |
| 4qMB157 | 4 | 157,447,597 | 157,447,597 | chr4:157447597-157447597 | chr4:157309442-157309442 |
| 4qMB179 | 4 | 179,727,852 | 179,729,814 | chr4:179727852-179729814 | chr4:179589697-179591659 |
| 5pMB31 | 5 | 31,080,349 | 31,082,443 | chr5:31080349-31082443 | chr5:31080349-31082443 |
| 5pMB35 | 5 | 35,452,161 | 35,454,297 | chr5:35452161-35454297 | chr5:35452161-35454297 |
| 5qMB113 | 5 | 113,290,806 | 113,292,730 | chr5:113290806-113292730 | chr5:113290806-113292730 |
| 5qMB123 | 5 | 123,245,996 | 123,247,944 | chr5:123245996-123247944 | chr5:123245996-123247944 |
| 6pMB45 | 6 | 45,850,117 | 45,852,150 | chr6:45850117-45852150 | chr6:45850117-45852150 |
| 7pMB42 | 7 | 42,243,941 | 42,245,949 | chr7:42243941-42245949 | chr7:42437226-42439234 |
| 7qMB152 | 7 | 152,697,552 | 152,699,497 | chr7:152697552-152699497 | chr7:152890837-152892782 |
| 7qMB83 | 7 | 83,132,176 | 83,134,117 | chr7:83132176-83134117 | chr7:83325461-83327402 |
| 9qMB100 | 9 | 100,675,660 | 100,675,660 | chr9:100675660-100675660 | chr9:102635926-102635926 |
| 11pMB20 | 11 | 20,226,825 | 20,228,784 | chr11:20226825-20228784 | chr11:20226825-20228784 |
| 11pMB23 | 11 | 23,520,249 | 23,522,237 | chr11:23520249-23522237 | chr11:23520249-23522237 |
| 13qMB31 | 13 | 31,082,858 | 31,084,774 | chr13:31082858-31084774 | chr13:31082858-31084774 |
| 13qMB64 | 13 | 64,790,190 | 64,792,122 | chr13:64790190-64792122 | chr13:64790190-64792122 |
| 13qMB67 | 13 | 67,866,542 | 67,866,542 | chr13:67866542-67866542 | chr13:67866542-67866542 |
| 13qMB69 | 13 | 69,958,755 | 69,960,759 | chr13:69958755-69960759 | chr13:69958755-69960759 |
| 14qMB85 | 14 | 85,990,054 | 85,992,114 | chr14:85990054-85992114 | chr14:85990054-85992114 |
| 15qMB35 | 15 | 35,863,258 | 35,865,250 | chr15:35863258-35865250 | chr15:35863258-35865250 |
| 16qMB53 | 16 | 53,125,682 | 53,127,694 | chr16:53125682-53127694 | chr16:53125682-53127694 |
| 17qMB66 | 17 | 66,215,066 | 66,217,016 | chr17:66215066-66217016 | chr17:66215066-66217016 |
| 18qMB34 | 18 | 34,301,883 | 34,303,943 | chr18:34301883-34303943 | chr18:34301883-34303943 |
| 18qMB47 | 18 | 47,987,485 | 47,989,232 | chr18:47987485-47989232 | chr18:47987485-47989232 |
| 18qMB60 | 18 | 60,706,873 | 60,706,873 | chr18:60706873-60706873 | chr18:60706873-60706873 |
| 18qMB67 | 18 | 67,222,650 | 67,224,357 | chr18:67222650-67224357 | chr18:67222650-67224357 |
