# Supplementary material for: Autosomal Resequence Data Reveal Late Stone Age Signals of Population Expansion in Sub-Saharan African Foraging and Farming Populations
Source: PLoS One. 2009 Jul 29;4(7):e6366. doi: 10.1371/journal.pone.0006366 (PMC2712685; doi:10.1371/journal.pone.0006366)
Supplement: Table S2 — Bayes factors for 3-dimensional 95% credible region inferred by ABC. (0.02 MB DOC) [file pone.0006366.s003.doc]

**Table S2**

| **Bayes factors for 3-dimensional 95% credible region inferred by ABC.** | | |
| --- | --- | --- |
| *Population* | *Bayes’ Factors (K)* | |
|  | *Rozas’ R_2_* | *Tajima’s D* |
| BIA | 70 | 61 |
| SAN | 67 | 70 |
| MAN | 67 | 70 |
| YOR | 57 | 57 |
