# Supplementary material for: Autosomal Resequence Data Reveal Late Stone Age Signals of Population Expansion in Sub-Saharan African Foraging and Farming Populations
Source: PLoS One. 2009 Jul 29;4(7):e6366. doi: 10.1371/journal.pone.0006366 (PMC2712685; doi:10.1371/journal.pone.0006366)
Supplement: Figure S1 — Validation results for the ABC procedure. Comparison of median values from joint posterior distributions for times of onset of growth (left column) and growth rates (right column). Median values (dotted vertical lines) inferred from 5 simulated datasets, each comprising 20 autosomal loci, are compared with known model parameters (solid vertical lines). ABC was employed with Rozas' R2 (upper row), Tajima's D (middle row), and both summary statistics jointly (bottom row). The number of segregating sites S was used to constrain θ in all cases. See text for details. (0.14 MB DOC) [file pone.0006366.s004.doc]

**Fig. S1**


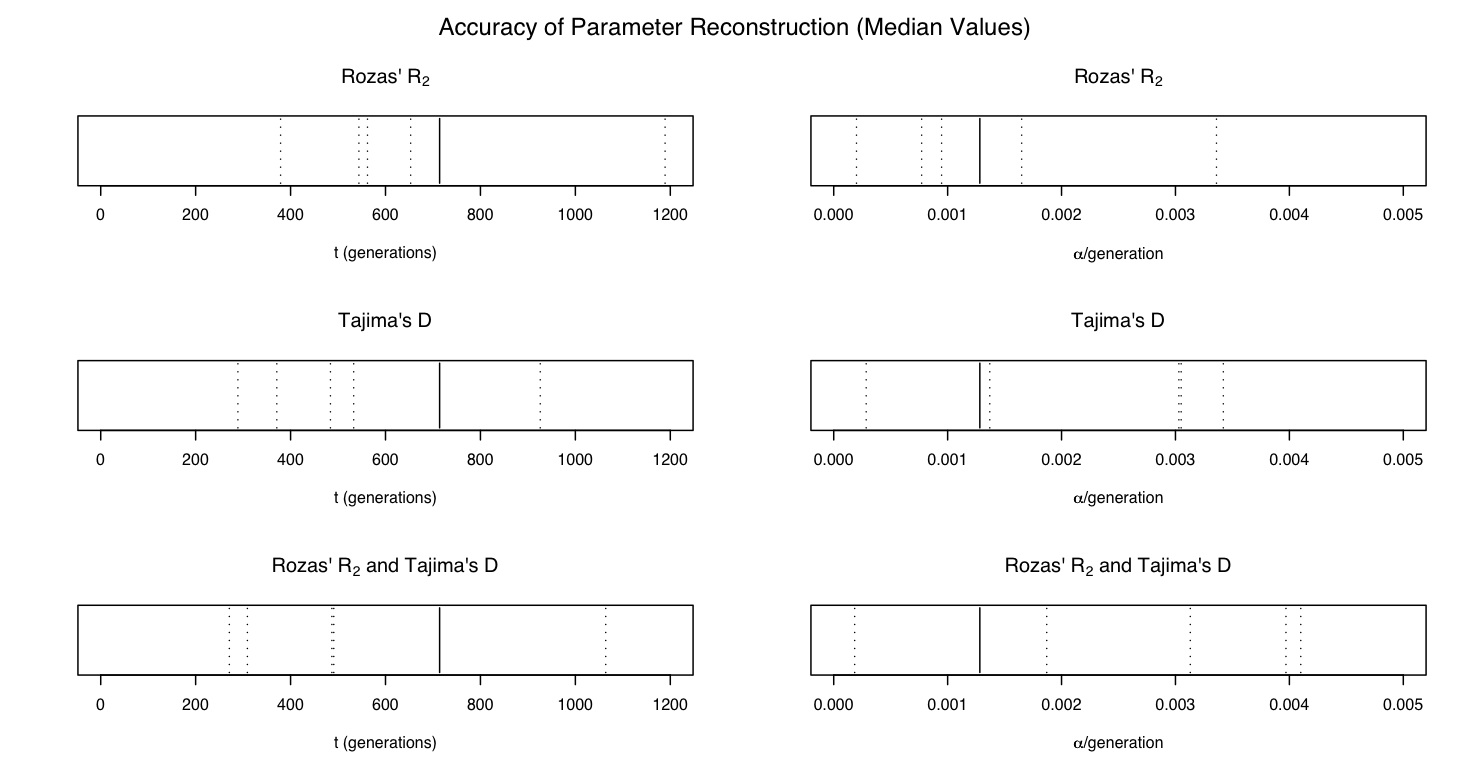


**Validation results for the ABC procedure.** Comparison of median values from joint posterior distributions for times of onset of growth (left column) and growth rates (right column). Median values (dotted vertical lines) inferred from 5 simulated datasets, each comprising 20 autosomal loci, are compared with known model parameters (solid vertical lines). ABC was employed with Rozas’ *R_2_* (upper row), Tajima’s *D* (middle row), and both summary statistics jointly (bottom row). The number of segregating sites *S* was used to constrain *θ* in all cases. See text for details.
