# Supplementary material for: Autosomal Resequence Data Reveal Late Stone Age Signals of Population Expansion in Sub-Saharan African Foraging and Farming Populations
Source: PLoS One. 2009 Jul 29;4(7):e6366. doi: 10.1371/journal.pone.0006366 (PMC2712685; doi:10.1371/journal.pone.0006366)
Supplement: Figure S2 — Validation results for the ABC procedure. Comparison of 95% credible region of joint posterior distributions for times of onset of growth (left column) and growth rates (right column). Lower confidence bounds, median values and upper confidence bounds inferred from 5 simulated datasets, each comprising 20 autosomal loci, are compared with known model parameters (solid vertical lines). ABC was employed with Rozas' R2 (upper row), Tajima's D (middle row), and both summary statistics jointly (bottom row). The number of segregating sites S was used to constrain θ in all cases. See text for details. (0.18 MB DOC) [file pone.0006366.s005.doc]

**Fig. S2**


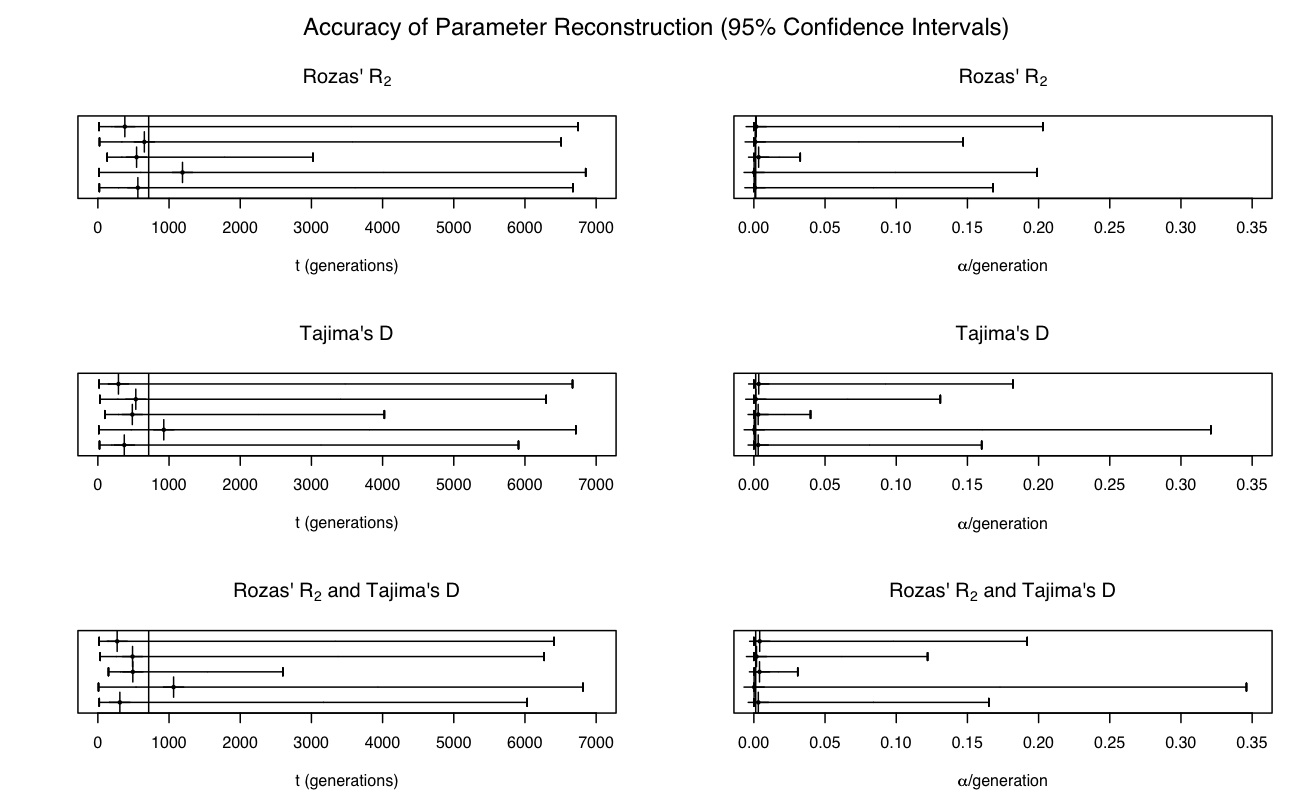


**Validation results for the ABC procedure.** Comparison of 95% credible region of joint posterior distributions for times of onset of growth (left column) and growth rates (right column). Lower confidence bounds, median values and upper confidence bounds inferred from 5 simulated datasets, each comprising 20 autosomal loci, are compared with known model parameters (solid vertical lines). ABC was employed with Rozas’ *R_2_* (upper row), Tajima’s *D* (middle row), and both summary statistics jointly (bottom row). The number of segregating sites *S* was used to constrain *θ* in all cases. See text for details.
