# Supplementary material for: Autosomal Resequence Data Reveal Late Stone Age Signals of Population Expansion in Sub-Saharan African Foraging and Farming Populations
Source: PLoS One. 2009 Jul 29;4(7):e6366. doi: 10.1371/journal.pone.0006366 (PMC2712685; doi:10.1371/journal.pone.0006366)
Supplement: Figure S5 — Effect of recent cryptic structure (∼150 years ago) on Rozas' R2 and Tajima's D in a two-deme splitting model. Circles indicate mean values; dotted lines indicate 95% credible regions. (0.10 MB DOC) [file pone.0006366.s008.doc]

**Fig. S5**


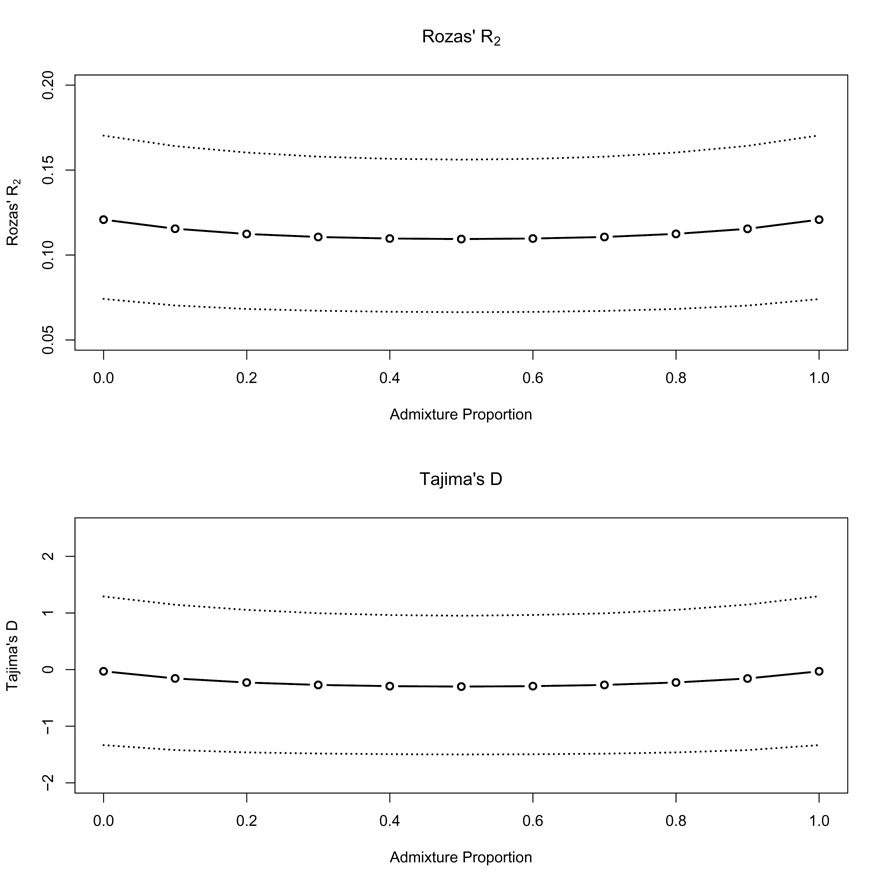


**Effect of recent cryptic structure (~150 years ago) on Rozas’ *R_2_* and Tajima’s *D* in a two-deme splitting model.** Circles indicate mean values; dotted lines indicate 95% credible regions.
