# Supplementary material for: Autosomal Resequence Data Reveal Late Stone Age Signals of Population Expansion in Sub-Saharan African Foraging and Farming Populations
Source: PLoS One. 2009 Jul 29;4(7):e6366. doi: 10.1371/journal.pone.0006366 (PMC2712685; doi:10.1371/journal.pone.0006366)
Supplement: Figure S6 — Time progression showing the expectation of Rozas' R2 following onset of growth. Haploid loci (circles) respond more quickly to growth (i.e., values of Rozas' R2 approaching zero) than autosomal loci (triangles). (0.08 MB DOC) [file pone.0006366.s009.doc]

**Fig. S6**


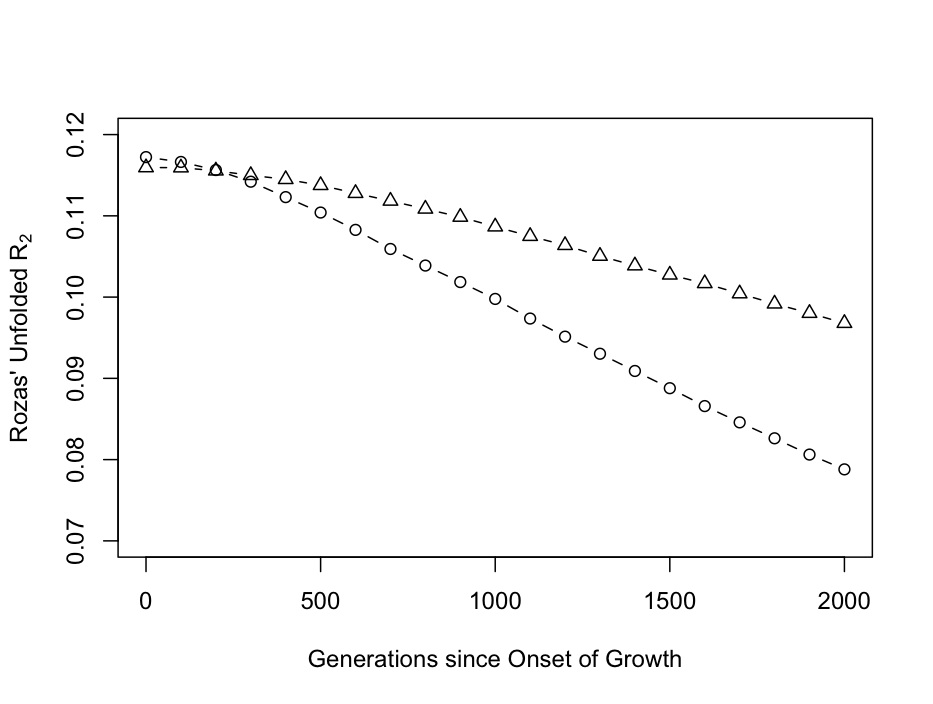


**Time progression showing the expectation of Rozas’ *R_2_* following onset of growth**. Haploid loci (circles) respond more quickly to growth (i.e., values of Rozas’ *R_2_* approaching zero) than autosomal loci (triangles).
