# Supplementary material for: Autosomal Resequence Data Reveal Late Stone Age Signals of Population Expansion in Sub-Saharan African Foraging and Farming Populations
Source: PLoS One. 2009 Jul 29;4(7):e6366. doi: 10.1371/journal.pone.0006366 (PMC2712685; doi:10.1371/journal.pone.0006366)
Supplement: Figure S7 — Time progression showing the expectation of Tajima's D following onset of growth. Haploid loci (circles) respond more quickly to growth (i.e., negative values of Tajima's D) than autosomal loci (triangles). (0.08 MB DOC) [file pone.0006366.s010.doc]

**Fig. S7**


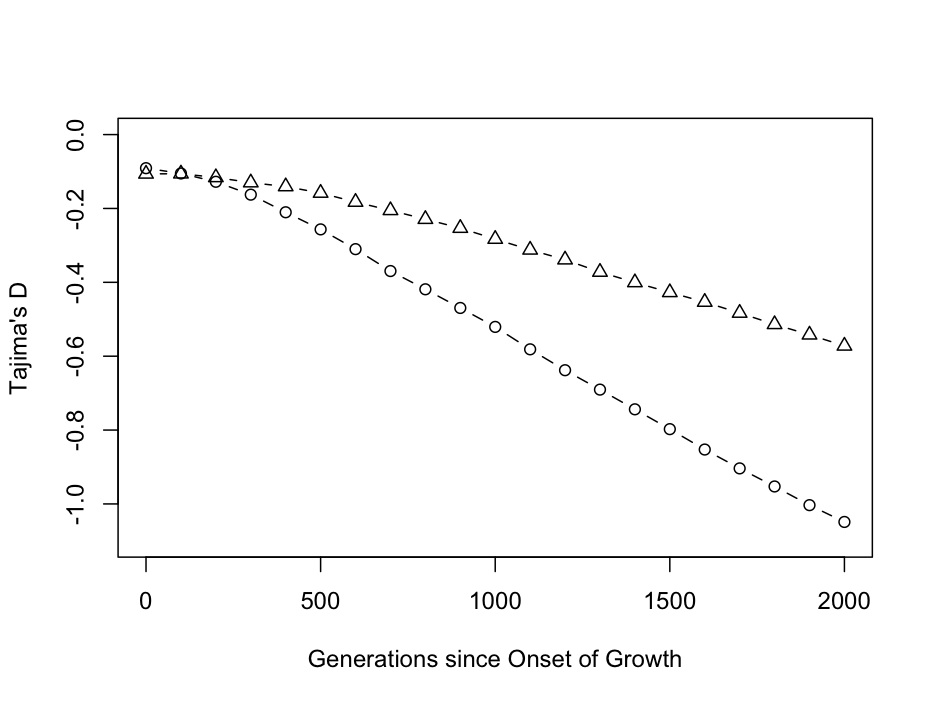


**Time progression showing the expectation of Tajima’s *D* following onset of growth**. Haploid loci (circles) respond more quickly to growth (i.e., negative values of Tajima’s *D*) than autosomal loci (triangles).
